# Supplementary material for: A Single Dose of the Deactivated Rabies-Virus Vectored COVID-19 Vaccine, CORAVAX, Is Highly Efficacious and Alleviates Lung Inflammation in the Hamster Model
Source: Viruses. 2022 May 24;14(6):1126. doi: 10.3390/v14061126 (PMC9229041; doi:10.3390/v14061126)
Supplement: Supplementary file 1 [file viruses-14-01126-s001.zip › viruses-1670362-SI.pdf]

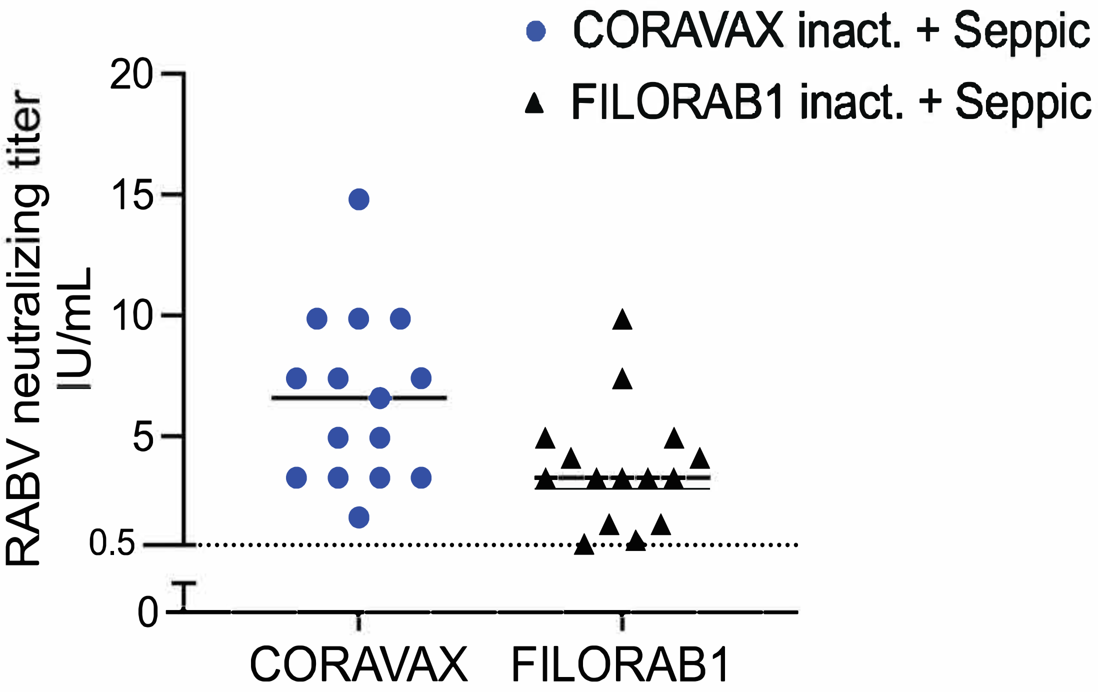

Supplementary Figure S1

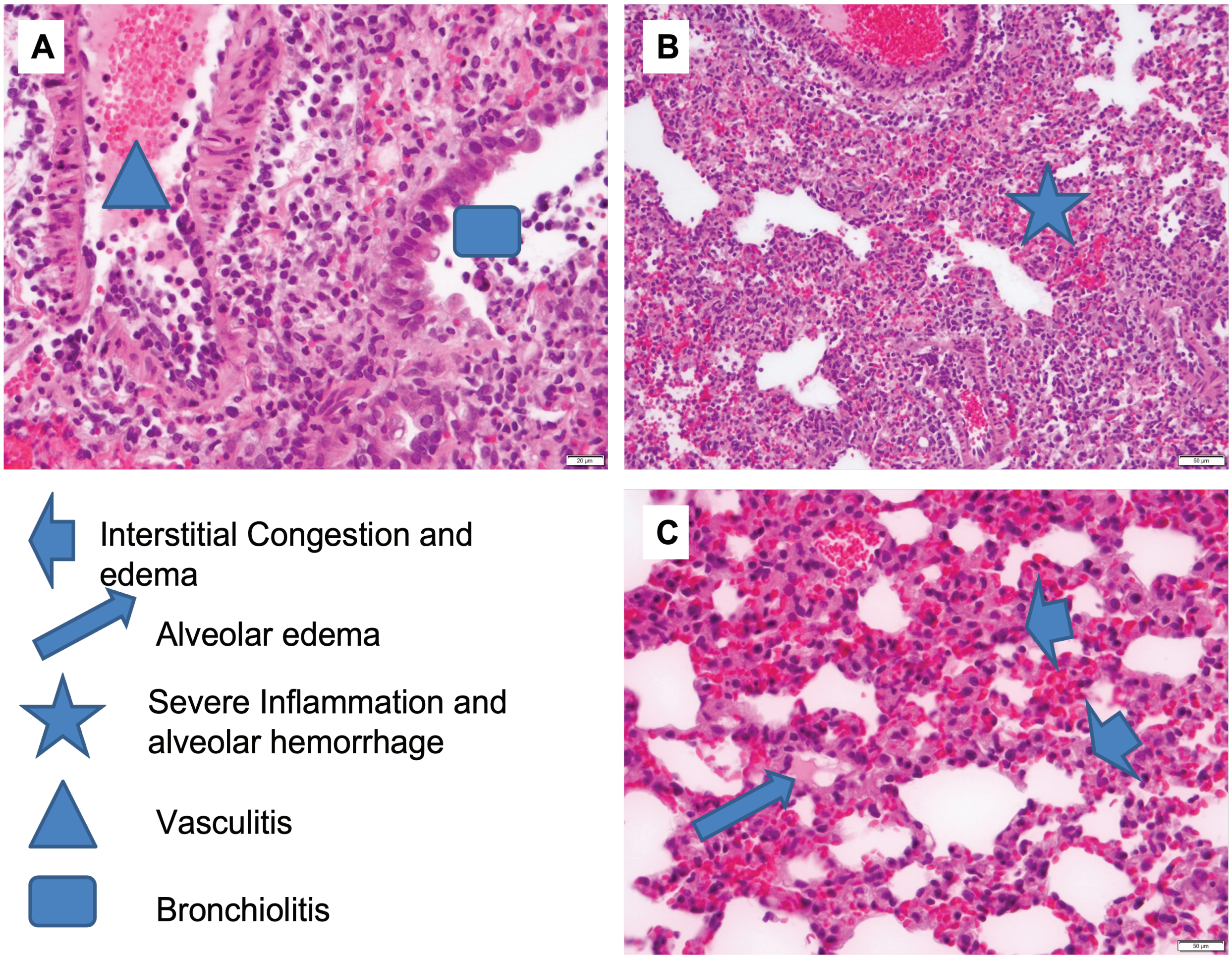

Supplementary Figure S2

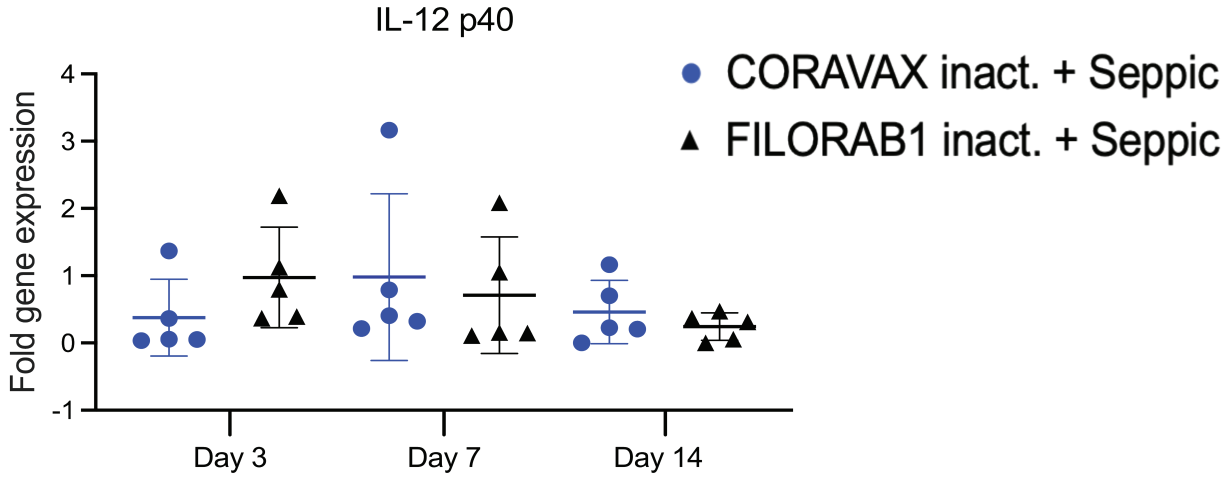

Supplementary Figure S3

Table S1. Semi-quantitative histopathological scoring criteria.

**Categories**	**Score criteria**	**Score (Total score=27 per lung)[a]**
General evaluation (G)	Assessment percentage of lung damage area under 4x objective lens	0 none<br>1 focal lung inflammation within one lobe or <30% of total lung cutting area[b]<br>2 diffuse lung inflammation within one lobe or involving 30-70% total lung cutting area<br>3 diffuse lung inflammation involving more than one lobe or 70% total lung cutting area
Bronchioles (B)	Assessing appearance and severity of peribronchiolar infiltration, intrabronchiolar wall infiltration and bronchiolar epithelial cell death/desquamation	0 none<br>1 peribronchiolar infiltration only<br>2 peribronchiolar and epithelial infiltration (mild to moderate)<br>3 severe bronchiolar wall infiltration and epithelium desquamation
Alveoli (A)	Assessing the appearance, scope involved, and severity of alveolar septal infiltration, alveolar space infiltration, alveolar space exudation and hemorrhage	0 none<br>1 only alveolar wall thickening (interstitial congestion and edema), mild interstitial pneumonitis (<30%)<br>2 alveolar wall infiltration (interstitial pneumonitis), exudation or hemorrhage involving 30-70% of lung cutting area<br>3 diffuse alveolar infiltration (interstitial pneumonitis), exudation or hemorrhage involving >70% of lung cutting area
Vasculature (V)	Severity of vasculature inflammation	0 none,<br>1 only perivascular edema and or perivascular infiltration<br>2 mild infiltration within the vessel wall without endothelium infiltration<br>3 severe vasculitis

a Total score = the scores of general assessment × the scores accumulated from individual category assessment b More than one lung lobes should be examined for histological assessment, if only one lobe were studied, percentage of the total lung cutting area would be used for scoring. Adapted from Lee et al. 2020 [1].

1. Lee AC*, et al.* Oral SARS-CoV-2 Inoculation Establishes Subclinical Respiratory Infection with Virus Shedding in Golden Syrian Hamsters. *Cell Rep Med* **1**, 100121 (2020).

Table S2. List of cytokine/chemokine qPCR primers and probes sequences.

Type of Multiplex	Cytokine/Chemokine	Reporter Dye		FW Primer 5' to 3'	REV Primer 5' to 3'	Probe 5' to 3'
5-Plex	γ-actin	VIC	MGB	ACA GAG AGA AGA TGA CGC AGA TAA TG	GCC TGA ATG GCC ACG TAC A	TTG AAA CCT TCA ACA CCC CAG CC
	IFN-γ	FAM	MGB	TGT TGC TCT GCC TCA CTC AGG	AAG ACG AGG TCC CCT CCA TTC	TGG CTG CTA CTG CCA GGG CAC ACT C
	CCL17	ABY	QSY	CGA GTG CTG CCT GGA GAT C	TGA TGG CCT TCT TCA CAT GC	TGTGACTGTCCAGGGCAGGTCCA
	CCL3	JUN	QSY	GGT CCA AGA GTA CGT CGC TG	GAG TTG TGG AGG TGG CAA GG	JIRA-55614; Thermo Fisher generated sequence
	CCL5	ALEXA680	QSY	TCA GCT TGG TTT GGG AGC AA	TGA AGT GCT GGT TTC TTG GGT	JIRA-55614;Thermo Fisher generated sequence
5-Plex	γ-actin	VIC	MGB	ACA GAG AGA AGA TGA CGC AGA TAA TG	GCC TGA ATG GCC ACG TAC A	TTG AAA CCT TCA ACA CCC CAG CC
	CXCL10	FAM	MGB	TAC GTC GGC CTA TGG CTA CT	TTG GGG ACT CTT GTC ACT GG	APH6E47; Thermo Fisher generated sequence
	IFN-α	ABY	QSY	TCG TTG CTG CTC CCG TAG TC	ATG GAT CCC GCT GCA ATT C	TGGGTGCTGTGGGCTTCACTGG
	IL-10	JUN	QSY	AGC TGG ACA ACA TAC TAC TCA CTG A	GGT TTG GCA ACC CAA GTA ACC	CTCCTTACTGCAGGACTTTAAG
	IL-12-p40	ALEXA680	QSY	AAT GCG AGG CAG CAA ATT ACT C	CTG CTC TTG ACG TTG AAC TTC AAG	CCT GCT GGT GGC TGA CTG CAA TCA
5-Plex	γ-actin	VIC	MGB	ACA GAG AGA AGA TGA CGC AGA TAA TG	GCC TGA ATG GCC ACG TAC A	TTG AAA CCT TCA ACA CCC CAG CC
	IL-17	FAM	MGB	AGGAACCTTGGAGAGATGGT	AGACAGGCTTCTCTTGTTGG	GCCCAGCTGAGGAACAGCTCTGAGCCAGCCAAGAG
	IL-2	ABY	QSY	GTG CAC CCA CTT CAA GCT CTA A	AAG CTC CTG TAA GTC CAG CAG TAA C	AGGAAACCCAGCAGCACCTCGAGC
	IL-21	JUN	QSY	GGA CAG TGG CCC ATA AAA CAA G	TTC AAC ACT GTC TAT AAG ATG ACG AAG TC	CAA GGG CCA GAT CGC CTC CTG ATT
	IL-4	ALEXA680	QSY	ACA GAA AAA GGG ACA CCA TGC A	GAA GCC CTG CAG ATG AGG TCT	AGA CGC CCT TTC AGC AAG GAA GAA CTC C
4-Plex	γ-actin	VIC	MGB	ACA GAG AGA AGA TGA CGC AGA TAA TG	GCC TGA ATG GCC ACG TAC A	TTG AAA CCT TCA ACA CCC CAG CC
	IL-6	FAM	MGB	CCT GAA AGC ACT TGA AGA ATT CC	GGT ATG CTA AGG CAC AGC ACA CT	AGAAGTCACCATGAGGTCTACTCGGCAAAA
	TGF-β1	ABY	QSY	GGC TAC CAC GCC AAC TTC TG	GAG GGC AAG GAC CTT ACT GTA CTG	CCC TGT CCC TAC ATT TGG AGC CTG GA
	TNF-α	JUN	QSY	TGA GCC ATC GTG CCA ATG	AGC CCG TCT GCT GGT ATC AC	CGG CAT GTC TCT CAA AGA CAA CCA G

Supplementary Table S2
